# Long non-coding RNA n326322 promotes the proliferation and invasion in nasopharyngeal carcinoma

**DOI:** 10.18632/oncotarget.22828

**Published:** 2017-12-01

**Authors:** Mingyu Du, Teng Huang, Jing Wu, Jia-Jia Gu, Nan Zhang, Kai Ding, Lu-Xi Qian, Zhi-Wei Lu, Wen-Jun Zhang, Xiao-Kang Tian, Xia He, Li Yin

**Affiliations:** ^1^ Xuzhou Medical University, Xuzhou, Jiangsu, China; ^2^ Department of Radiation Oncology, Jiangsu Province Hospital of TCM, Nanjing, Jiangsu, China; ^3^ The Fourth Clinical Medical College of Nanjing Medical University, Nanjing, Jiangsu, China; ^4^ Jiangsu Cancer Hospital and Jiangsu Institue of Cancer Research and Nanjing Medical University Affiliated Cancer Hospital, Nanjing, Jiangsu, China

**Keywords:** nasopharyngeal carcinoma, lncRNA-n326322, invasion

## Abstract

Long non-coding RNAs (lncRNAs) have been reported to perform significant roles in cancer development and progression. Our research has found that a novel lncRNA n326322 was higher in nasopharyngeal carcinoma (NPC) cells. Moreover, the gain and loss of functional approaches revealed that the overexpression of lncRNA-n326322 promoted NPC cell proliferation and invasion, whereas the downregulation of lncRNA-n326322 suppressed cell proliferation and invasion. Further experiments demonstrated that potential mechanism may be associated with the activation of PI3K/AKT and ERK/MAPK pathways. Taken together, these results indicate that lncRNA-n326322 is associated with tumorigenesis of NPC.

## INTRODUCTION

Nasopharyngeal carcinoma (NPC) is a common aggressive carcinoma arising from the nasopharynx epithelium [1]. It is vastly prevalent in Southeast China and some other Asian countries [1]. Thanks to the development of radiation therapy and adjuvant chemotherapy, the average 5-year survival rate for NPC patients has been greatly improved [1–4]. However, tumor recurrence and distant metastases are still the main obstacles for patient survival [5–6]. Elucidating the molecular mechanisms of pathogenesis to find notable therapeutic strategies for NPC patients is urgently needed.

Long noncoding RNAs (lncRNAs) are a class of transcripts longer than 200 nucleotides in length with limited or no protein-coding functions [7]. Accumulating evidence indicates that dysregulated lncRNA expression levels are correlated with cancer progression and development by modulating gene expression in transcriptional or posttranscriptional levels [8–13]. For example, lncRNA urothelial cancer-associated 1 (UCA1) has been shown to overexpress in bladder cancer, and the high expression of UCA1 frequently enhanced the invasive and migratory capacity of bladder cancer cells [13]. Recent studies have also reported that lncRNA metastasis-associated lung adenocarcinoma transcript 1 (MALAT1) could promote epithelial-to-mesenchymal transition (EMT) and metastasis in many cancers, including renal cell carcinoma, oral squamous cell carcinoma, and endometrioid endometrial carcinoma [14–16]. However, only a few studies exist, focusing on dysregulated lncRNAs in NPC and their functions that remain largely unknown [17–19].

In this study, we have identified a new lncRNA-n326322 (Noncode V3 ID: n326322), which was upregulated in NPC cell lines. We also found that lncRNA-n326322 could regulate cell proliferation through PI3K/AKT pathway and promote cell invasion through ERK/MAPK pathway in NPC.

## RESULTS

### lncRNA-n326322 is upregulated and associated with tumorigenesis of NPC

**Figure 1:**
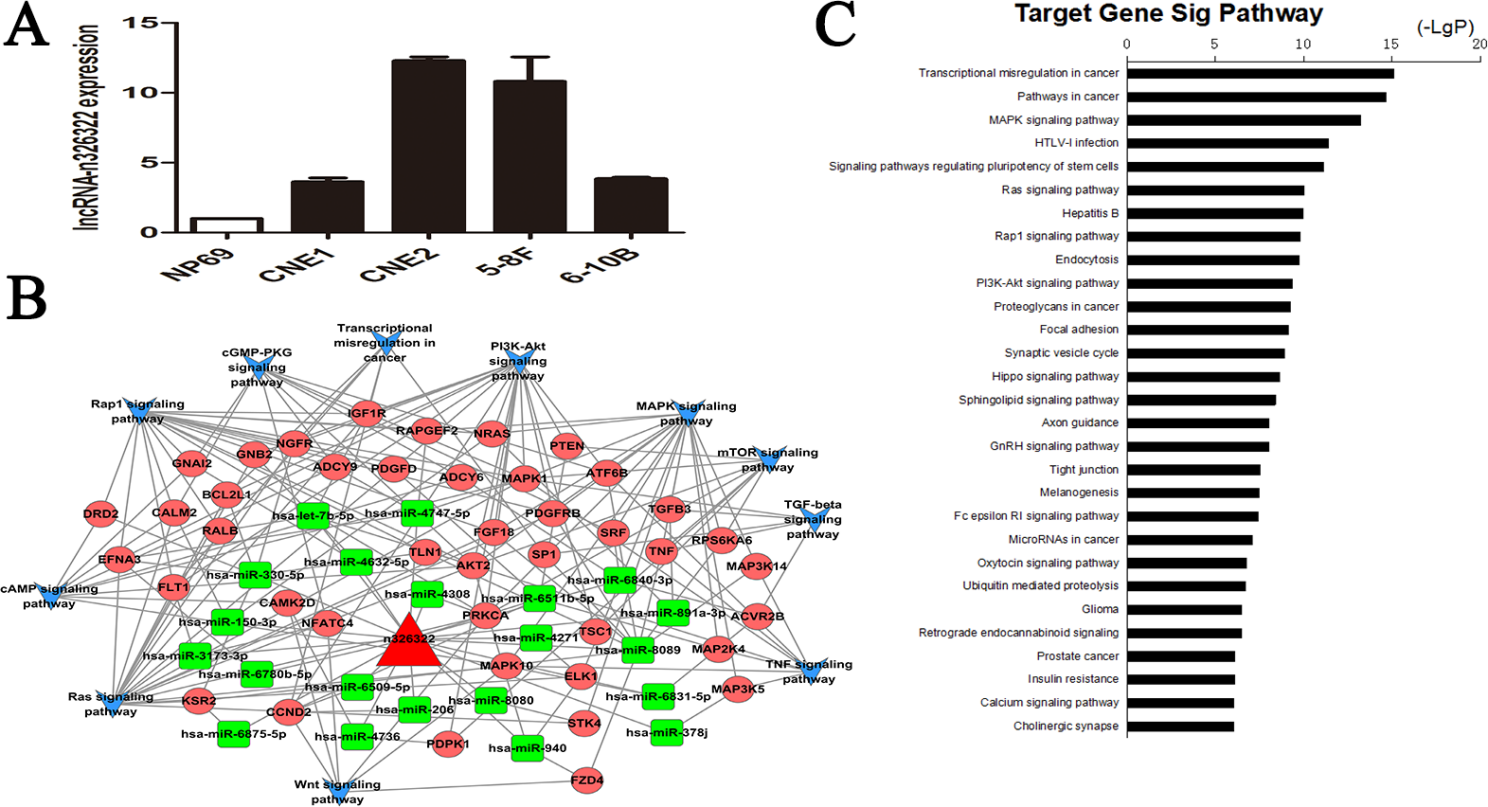
lncRNA-n326322 is upregulated in NPC cell lines (**A**) Expression of lncRNA-n326322 in NPC cell lines. (**B**) Global view of ceRNA network consists of the miRNAs, miRNA targets and lncRNA-n326322. (**C**) Significantly enriched KEGG pathway of miRNA targets in the ceRNA network.

The expression levels of lncRNA-n326322 in NPC cell lines were analyzed by qRT-PCR, and the results showed that lncRNA-n326322 expression was significantly higher in NPC cells than in immortalized NP69 cells (Figure 1A). To predict the potential biological functions of the novel lncRNA-n326322 in tumor formation, we employed bioinformatic analyses based on some emerging themes. One of these themes is that lncRNAs perform their regulatory functions partly through interaction with RNA-binding proteins (RBPs). Moreover, with the rapid development of high-throughput sequencing of RNAs, it has provided powerful ways to map the interaction of lncRNAs and RBPs [20, 21]. We used RBPDB (http://rbpdb.ccbr.utoronto.ca/), a database of RNA-binding specificities, to search for the possible RBPs that could be sequence complementary with lncRNA-n326322. As shown in Table 1, we found a total of 16 RBPs that could match to lncRNA-n326322, including RBMY1A1, EIF4B, and Pum2, which have been reported to have an important influence on tumor progression [22–24]. In addition, accumulating studies have demonstrated a competitive endogenous RNA (ceRNA) hypothesis stating that lncRNA might serve as a molecular sponge to modulate miRNA expression [25]. We employed database software to determine conceivable miRNAs that could bind to lncRNA-n326322 and eventually found a total of 30 paired miRNAs. Then, we performed computational methods to confirm these possible miRNA targets. Here, we integrated lncRNA-n326322, its paired miRNAs, and miRNA targets to construct a global ceRNA network (Figure 1B). To further explore this interaction network, we used the Kyoto Encyclopedia of Genes and Genomes (KEGG) pathways to analyze these targets. Interestingly, we found that these lncRNA-associated ceRNA targets are involved in some KEGG pathways including cancer pathways and some other signaling pathways (Figure 1C). Among these enriched signaling pathways, such as the MAPK and Ras signaling pathways, as well as the PI3K–Akt signaling pathway, have been identified to contribute to the tumorigenesis of NPC [26–28].

As discussed above, lncRNA-n326322 is upregulated and associated with tumorigenesis of NPC.

### lncRNA-n326322 promotes NPC cell proliferation

**Figure 2:**
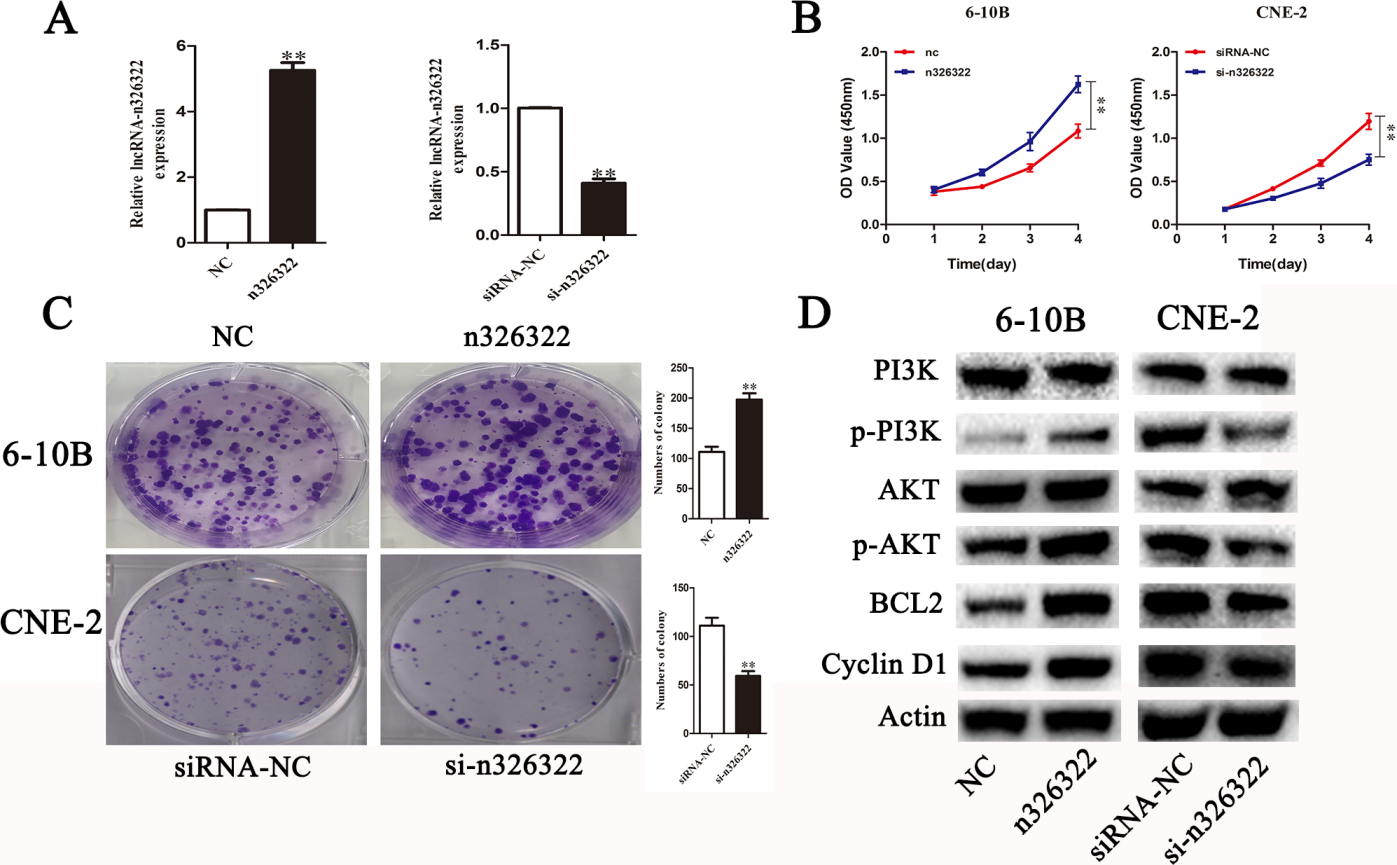
LncRNA-n326322 promotes NPC cell proliferation (**A**) The efficiency of siRNA-n326322 and n326322 overexpression was determined by qRT-PCR. ^**^P < 0.05 compared with control. (**B**) CCK8 assays indicated that the upregulation of lncRNA-n326322 promoted 6-10B cell viability, and the downregulation of lncRNA-n326322 inhibited the proliferation of CNE2 cells. ^**^P < 0.05 compared with control. (**C**) Colony formation was used to analyze cell viability of transfected 6-10B and CNE-2 cells. ^**^P < 0.05 compared with control. (**D**) The Western blot analysis showed the expression of the PI3K/AKT signaling pathways controlled by lncRNA-n326322.

To investigate the effects of lncRNA-n326322 on the proliferation of NPC cells, gain and loss of functional approaches were performed. As shown in Figure 1A, NPC cell lines of CNE-2 expressed the highest levels of n326322, whereas 6-10B showed the lowest levels of n326322 among these tested NPC cell lines. Thus, we selected the CNE-2 and 6-10B cells for further studies. The expression levels of lncRNA-n326322 in 6-10B cells or CNE-2 cells were analyzed by qRT-PCR after transfection with pcDNA-n326322 or n326322-siRNA. In comparison with the negative control, the 6-10B cells transfected with n326322 overexpression vector evidently exhibited an increased n326322 mRNA level, whereas the knockdown efficiency of lncRNA-n326322 in CNE-2 cells was nearly 60%. Based on the growth curves determined by Cell Counting Kit-8 (CCK-8) assays, the forced expression of lncRNA-n326322 dramatically improved cell viability, whereas suppression of n326322 had the opposite effect in CNE-2 cells (Figure 2B). We also used colony formation assays to confirm our findings. As shown in Figure 2C, lncRNA-n326322 substantially increased the colony formation efficiency in NPC cell lines.

To understand the mechanism of n326322 in promoting cell proliferation, Western blot analysis was performed. Based on our previous bioinformatic analyses, we believed that PI3K/Akt may be involved in NPC progression. The PI3K/Akt signaling pathway has been reported to possess important roles in cell growth and proliferation [28, 29]. Interestingly, dozens of studies have demonstrated that the PI3K/Akt signaling pathway is one of the downstream signals activated by lncRNAs [30, 31]. Jing et al. illustrated that the knockdown of HOTAIR suppressed the cell proliferation by serving as a molecular sponge of miR-326 via the PI3K/Akt signaling pathway in human glioma cells [30]. Additionally, Zhu et al. showed that lncRNA HULC, an oncogene in gliomas, promoted angiogenesis by up-regulating ESM-1 expression through the PI3K/Akt signaling pathway [31]. In this present study, overexpression of lncRNA-n326322 significantly improved the levels of phosphorylated PI3K and Akt, whereas downregulation of lncRNA-n326322 suppressed these protein levels. However, the expression levels of PI3K and Akt were stable (Figure 2D). The expression levels of BCL2 and Cycling D1, known as important downstream components of PI3K/Akt signaling pathway, were also detected by the Western blot analysis [31]. The levels of BCL2 and Cycling D1 were improved due to the restoration of lncRNA-n326322 and depressed owing to the knockdown of lncRNA-n326322. All these results reveal that n326322 regulates NPC cell proliferation via PI3K/Akt signaling pathways.

### lncRNA-n326322 promotes NPC cell invasion and migration

**Figure 3:**
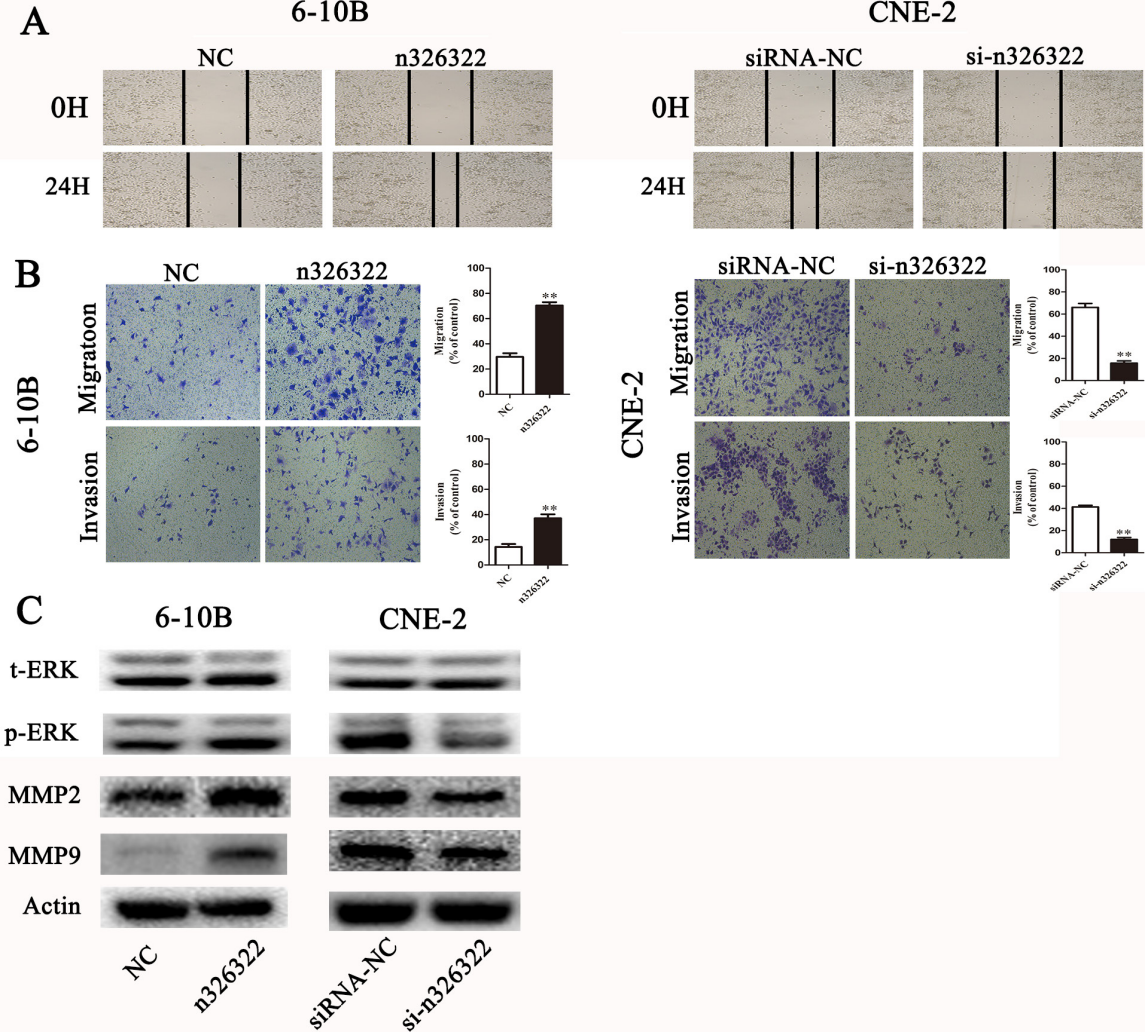
lncRNA-n326322 promotes NPC cell invasion and migration (**A**) The migration capability of NPC cells regulated by lncRNA-n326322 was determined by wound-healing assays. (**B**) Transwell assay with or without matrigel showed that upregulation of lncRNA-n326322 increased cell invasion and migration in 6-10B cells, whereas downregulation of lncRNA-n326322 suppressed cell invasion and migration capability in CNE-2 cells. ^**^P < 0.05 compared with control. (**C**) Western blot results demonstrated that the overexpression of lncRNA-n326322 induced the expression of p-ERK, MMP2 and MMP9, and the knockdown of lncRNA-n326322 inhibited the expression of p-ERK, MMP2 and MMP9.

To examine the promotion of the invasive behavior of NPC cells by lncRNA-n326322, wound-healing and transwell assays were used. Wound-healing assays demonstrated that exogenous expression of lncRNA-n326322 simulated the migratory potential of 6-10B cells, but knock-down of lncRNA-n326322 expression significantly delayed scratch healing in CNE-2 cells (Figure 3A). The transwell assay without matrigel also confirmed that upregulated lncRNA-n326322 improved cell migration, whereas downregulated lncRNA-n326322 inhibited this ability (Figure 3B). Furthermore, the transwell assay with matrigel showed that the number of invasive cells increased in n326322 overexpressing cells compared with the negative control cells, whereas the number of invasive cells with downregulated lncRNA-n326322 was decreased (Figure 3B).

According to our bioinformatic analyses, the ERK/MAPK pathway, which was often aberrantly activated in human cancers and led to enhanced cell invasiveness [32, 33], might take part in n326322-driven tumor invasion. We performed the Western blot analysis to examine the expression levels of upregulated or downregulated lncRNA-n326322 on the ERK/MAPK signaling pathway. As shown in Figure 3C, the protein levels of phosphorylated ERK were higher in lncRNA-n326322 overexpressing cells, and the opposite result was observed in lncRNA-n326322 knock-down cells, whereas no detectable changes were found in the total levels of ERK. It is widely acknowledged that matrix metalloproteinases (MMPs) are the vital downstream signaling components of the ERK/MAPK pathway [34, 35]. Particularly, upregulations of MMP2 and MMP9 are associated with NPC tumor progression and enhance the migration and invasion of NPC cells [36]. Here, we identified that the expression levels of MMP2 and MMP9 in lncRNA-n326322 overexpressing cells were reduced and increased in lncRNA-n326322 knock-down cells compared with those in the control cells. Hence, lncRNA-n326322 might promote the invasive and migratory capacities of NPC cells through the ERK/MAPK signaling pathways.

## DISCUSSION

Accumulating evidence has revealed that lncRNAs play important biological roles in human cancers, and that dysregulated lncRNAs are significantly related to the occurrence and development of tumors [37–40]. Several lncRNAs have also been identified in NPC development, indicating a poor prognosis [41–45]. In this present study, lncRNA-n326322 was overexpressed more in the NPC cell lines compared with NP69, and the bioinformatic analyses showed that lncRNA-n326322 might be associated with tumorigenesis of NPC.

lncRNA-n326322 (Noncode V3 ID: n326322; Noncode Gene ID: NONHSAG056983 and Noncode TRANSCRIPT ID: NONHSAT150079) is located in chromosome 1 (chr1: 175618208–175632895), and the mature lncRNA-n326322 transcript is 491 bp in length. In the present study, we used CCK8 and colony formation assays to confirm that the overexpression of lncRNA-n326322 in 6-10B cell lines promoted cell proliferation, whereas the downregulation of lncRNA-n326322 in CNE-2 cells significantly reduced cell growth. Then, we performed the Western blot analysis to investigate the possible cancer-related pathways that could increase cell viability. Many lncRNAs have been reported to promote tumorigenesis through the PI3K/Akt signaling pathway [30, 41]. We test the expression of p-PI3K and p-Akt and surprisingly found that they were markedly enhanced by exogenous expression of lncRNA-n32632 and reduced by downregulated expression of lncRNA-n326322, whereas no changes were detected in the expression of PI3K and Akt. Meanwhile, the expression levels of BCL2 and Cycling D1, the downstream molecules of PI3K/Akt signaling pathway, were also increased due to the restoration of lncRNA-n326322 and depressed owing to the knockdown of lncRNA-n326322. These lines of evidence indicated that lncRNA-n326326 promoted cell proliferation by activating the PI3K/Akt signaling pathway. However, the mechanism of how lncRNA-n326322 sensitized this pathway remains unclear. Interaction with RBP and CeRNA hypothesis are important mechanisms for lncRNAs to perform biological functions [20, 25]. On the other hand, through our established online software analysis, we uncovered some potential RBPs for lncRNA-n326322 targets. EIF4B, one of these RBPs, has been considered as an oncogene in tumor progression, and Shahbazian et al. showed that the mTOR/PI3K pathway converged on eIF4B [46, 47]. In this present study, lncRNA-n326322 might facilitate PI3K/Akt signaling by regulating the expression of EIF4B. On the other hand, through the bioinformatic analysis, we found that lncRNA-n326322 may improve the expression of FGF18 by serving as a molecular sponge to modulate miR-8089. FGF18 is a positive regulator of PI3K/Akt signaling pathways [48].

lncRNA-n326322 could activate invasion and migration of NPC cells. Western blot results revealed that the elevated invasion and migration by overexpression of lncRNA-n326322 was possibly linked to the activated ERK/MAPK pathway, which has been widely confirmed in cancers [49, 50]. MMP2 and MMP9, downstream of the ERK/MAPK pathway, are popular key enzymes for invasion and migration [51]. During our study, the data showed that upregulated lncRNA-n326322 induced the expression of p-ERK, MMP2, and MMP9; on the contrary, downregulated lncRNA-n326322 reduced the expression of p-ERK, MMP2, and MMP9. Although the precise mechanisms on how lncRNA-n326322 activated the ERK/MAPK signaling pathway are still unknown, a growing number of reports have thrown light on lncRNA-mediated regulation on this pathway [52, 53]. A recent study has reported that a new lncRNA HCG11-derived oncogenic growth through interaction with the RNA-binding protein IGF2BP1, contributes in facilitating the ERK/MAPK pathway in hepatocellular carcinoma [52]. Wang et al. also found that lncRNA UCA1 could function as a molecular sponge of miR-216b and activate the FGFR1/ERK signaling pathway in hepatocellular carcinoma [53]. In this present study, the underlying mechanisms that lncRNA-n326322 facilitate the ERK/MAPK signaling pathway may be also associated with RBPs and miRNAs. Our bioinformatic analysis provided some RBPs or miRNAs, such as eIF4B and miR-150, which have been proved to interact with the ERK/MAPK pathway [46, 47, 54]. However, further studies need to be performed to confirm whether these molecules are involved in lncRNA-mediated regulation on the ERK/MAPK signaling pathway.

**Figure 4:**
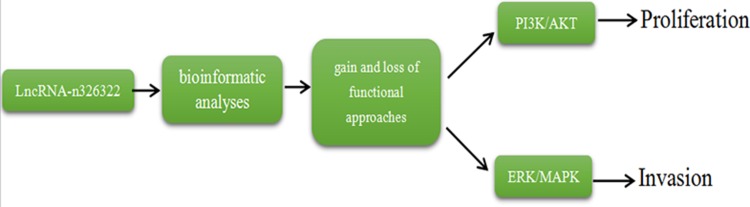
lncRNA-n326322 promotes the proliferation and invasion of NPC cells

In summary, we identified novel lncRNA-n326322 as an important mediator of proliferation and invasion of NPC cells by PI3K/Akt and ERK/MAPK signaling pathways (Figure 4) and provided a new insight into the mechanism of invasion and metastasis of NPC.

## MATERIALS AND METHODS

### Cell culture

A human immortalized nasopharyngeal epithelial cell line (NP69) and four NPC cell lines (CNE1, CNE2, 5–8F, 6–10B) originated from the Research Center of Clinical Oncology of the Affiliated Jiangsu Cancer Hospital, Nanjing Medical University, Nanjing, China. Cells were cultured in RPMI 1640 medium (Gibco, Grand Island, NY) containing 5% fetal bovine serum (Gibco, USA) and incubated at 37°C in the presence of 5% CO_2_.

### RNA extraction and quantitative real-time PCR assays

Total RNA was extracted using TRIzol reagent (Invitrogen, Carlsbad, CA) in accordance with the manufacturer's instructions. Then, 2 μg of total RNA was reversely transcribed into cDNA using M-MLV Reverse Transcriptase (Promega, Madison, WI, USA). Real-time PCR was performed using SYBR Green PCR master mix on the ABI7300 real-time PCR machine (Applied Bio-systems). The expression of n326322 and β-actin was examined using the following specific primers: 5′-TCATTCCACATCAAGCCCCG-3′ and 5′-TGGATATCGGGCTACAGGT-3′ and 5′-GGACTTCGAGCAAGAGATGG-3′ and 5′-AGCACTGTGTTGGCGTACAG-3′. Fold changes for n326322 expression levels were calculated using the 2^ΔΔCt^ method

### Cell transfection

siRNA-NC and siRNA-n326322 were synthesized by RiboBio (Guangzhou, China). The full length of n326322 was subcloned into the pcDNA3.1 plasmid, and the pcDNA3.1 empty vector was used as the control plasmid. CNE-2 and 6-10B cells were cultured in six-well plates for 24 h and transfected with siRNA-n326322 or n326322 using Lipofectamine 2000 (Invitrogen, Carlsbad, CA) according to the manufacturer's instructions. To determine the efficiency of siRNA-n326322 or n326322, the expression of n326322 was assessed with real-time PCR.

### Cell viability assay

Growth curves were determined by the CCK-8 according to the manufacturer's instructions. After the 48 h transfection, 3 × 10^3^ cells/well were seeded in 96-well plates with five replicate wells for each group. Cells were cultured for 1, 2, 3, or 4 days before 10 ml CCK-8 was added to the 100 μL of culture medium for each well and incubated at 37°C for 1 h according to the manufacturer's instructions. The optical density in each well was determined at a wavelength of 450 nm by a microplate reader. The experiment was repeated three times, and the data shown are the average of these three repeated measurements.

### Colony forming assay

To measure the colony-forming activity, CNE-2 and 6-10B cells were trypsinized and placed into a six-well plate (1 × 10^2^ cells/well) after transfection and cultured for 10 days. The colonies were fixed with paraformaldehyde and stained with 0.1% crystal violet. Then, the numbers of colonies were counted under an inverted microscope. All steps were performed in triplicate.

### Cell wound-healing assay

Wound-healing assay was conducted to examine the capacity of cell migration. CNE2 and 6-10B cells were cultured in six-well plates and transfected with siRNA or n326322, and then maintained for 48 h until confluency. A wound was created by manually scratching the surface of the plates with a 200-μL pipette tube, and the floated cells were removed by washing with PBS twice. Cell migration was monitored under an optical microscope with a magnification of 100×. Each experiment was performed in triplicate.

### Cell invasion assay

Matrigel-coated (BD Biosciences) membrane and migration assay with no matrigel membrane were used to assess cell invasion or migration ability according to the manufacturer's instructions. Transfected CNE-2 and 6-10B cells were cultured for 48 h. The cells were resuspended (2 × 10^4^ cells/well) in 200 μL of serum-free media and placed in the upper compartment of a chamber (Corning, NY, USA). Meanwhile, the bottom chamber was filled with 500 μL of RPMI-1640 and 20% fetal bovine serum. The cells that migrated or invaded the lower surface of the membrane were fixed with 4% paraformaldehyde and stained with crystal violet after 24 h of incubation. Then, five random fields of cells were counted under a light microscope at a magnification of 100×. All experiments were performed three times.

### Western blot analysis

Total cellular proteins were extracted from the transfected cells using modified RIPA buffer (Beyotime, Shanghai, China), and protein concentration was quantified using a BCA protein assay kit (Beyotime, Shanghai, China). A total of 15 mg of protein from each sample was separated using 10% sodium dodecyl sulfate polyacrylamide gel electrophoresis and then transferred to polyvinylidene difluoride membranes (Millipore, Billerica, MA). After blocking with BSA in Tris-buffered saline/Tween-20, The membranes were incubated with the indicated primary antibodies: anti-PI3K/p-PI3K (1:1000; Cell Signaling Technology, USA), anti-Akt/p-Akt (1:1000; Cell Signaling Technology, USA), anti-ERK/p-ERK (1:1000; Cell Signaling Technology, USA), anti-MMP9 (1:1000; Cell Signaling Technology, USA), and anti-β-actin(1:2000;Cell Signaling Technology, USA) at 4°C overnight and then incubated with the corresponding secondary antibodies at room temperature for 2 h. Immunoreactive bands were visualized using the ECL detection reagent (Millipore, Billerica, MA, USA). All data analyses were repeated thrice independently.

### Statistical analysis

All statistical analyses were conducted using Student's *t*-test and one-way analysis of variance with Graphpad 5.0 and SPSS 13.0. Data were expressed as mean ± standard deviation in at least three separate experiments. *P* < 0.05 was considered statistically significant.
